# Experimental phasing of serial femtosecond crystallography data

**DOI:** 10.1107/S2052252517012167

**Published:** 2017-09-01

**Authors:** Ilme Schlichting

**Affiliations:** a Max Planck Institute for Medical Research, Heidelberg, Germany

**Keywords:** serial femtosecond crystallography, anomalous signal, data quality, *de novo* phasing, XFELs

## Abstract

A synopsis of and prospects for *de novo* phasing using diffraction data collected at X-ray free-electron lasers are given.

X-ray free-electron lasers (XFELs) provide ultra-bright femtosecond X-ray pulses that are intense enough to enable data collection of small weakly scattering objects and short enough to outrun most radiation damage effects. These beam features allow not only to study highly radiation-sensitive systems such as metalloproteins and tiny crystals but also to capture fleeting reaction intermediates in time-resolved studies. They therefore expand the structural biologist’s toolbox by a great deal (Schlichting, 2015 ▸; Spence, 2017 ▸).

*De novo* phasing of XFEL data, however, remains problematic, for several reasons. First, there is the stochastic nature of the XFEL beam parameters (pulse and photon energy, including the spectral distribution of the latter, pointing, beam profile, *etc.*). Second, there is the serial femtosecond crystallography (SFX) data collection scheme that only once probes randomly oriented microcrystals intersecting the XFEL beam and gives a still image. Thus, it had long been doubted that the resulting integrated Bragg intensities are accurate enough for *de novo* phasing to be successful. A number of XFEL-specific phasing approaches have been suggested (Spence *et al.*, 2011 ▸; Son *et al.*, 2011 ▸; Ayyer *et al.*, 2016 ▸), but so far none have demonstrated success in *de novo* phasing of experimental data.

Nevertheless, indications that phasing might indeed work came from the observation of the weak anomalous signal of sulfur (Barends *et al.*, 2013 ▸), which was soon afterwards demonstrated by the first *de novo* phasing of lysozyme using the strong anomalous signal of bound gadolinium ions (Barends *et al.*, 2014 ▸). This was followed by using mercury for single isomorphous replacement with anomalous scattering (SIRAS) (Yamashita *et al.*, 2015 ▸, 2017 ▸), iodine substitutions in a detergent molecule to phase SFX data of the model membrane protein bacteriorhodopsin (Nakane *et al.*, 2016 ▸), exploiting the anomalous signal of endogenous sulfur atoms (Nakane *et al.*, 2015 ▸; Nass *et al.*, 2016 ▸; Batyuk *et al.*, 2016 ▸) or metal centres (Fukuda *et al.*, 2016 ▸) and inserting seleno­methio­nine in recombinantly expressed protein (Yamashita *et al.*, 2017 ▸; Hunter *et al.*, 2016 ▸). All these studies used model systems that diffract strongly to high resolution and relied on phasing approaches (SAD, SIR, SIRAS) that are commonly used at synchrotron sources. A novel approach, again using a well established model system, was demonstrated recently by exploiting a dual colour mode of the XFEL source SACLA delivering two XFEL pulses widely separated in photon energy simultaneously (Hara *et al.*, 2013 ▸), thereby enabling efficient SFX data collection for multi-wavelength anomalous dispersion (MAD) phasing while saving sample and beam time (Gorel *et al.*, 2017 ▸).

Given all this progress it would almost seem as if XFEL *de novo* phasing were coming of age. However, so far only one previously unknown structure has been revealed by *de novo* phasing of XFEL data. The structure of the mosquito larvicide BinAB was solved by multiple isomorphous replacement with anomalous scattering (MIRAS) using *in vivo* grown nanocrystals diffracting to better than 2.5 Å resolution (Colletier *et al.*, 2016 ▸). Indeed, having high-resolution data was important for all successful examples solved so far. In contrast to the atomic form factor which decreases strongly with scattering angle, the anomalous scattering signal *f*′′ does not depend on it. Therefore, in principle, the contribution of the anomalous signal increases with resolution. However, since high-resolution data are typically measured less accurately, using crystals that diffract strongly to high resolution makes a big difference for phasing. Indeed, in a systematic study describing selenium- and mercury-based anomalous phasing approaches published in this issue of *IUCrJ*, Yamashita *et al.* (2017 ▸) show that significantly higher multiplicity of measurements for individual reflections and thus many more diffraction images are needed when reducing the resolution of a mercury derivative dataset for SIRAS phasing from 1.5 Å to 2.6 Å (400000 patterns instead of 11000). Importantly, this large increase in multiplicity is required despite the fact that the data are excellent and very strong at 2.6 Å (unlike for systems that really stop diffracting at 2.6 Å). They partly attribute this to the generally poor quality of low-resolution SFX data.

Knowing whether one can solve a structure by SAD phasing and when to stop collecting more data because the anomalous signal in SAD phasing is high enough is still an active area of research [see, for example, Terwilliger *et al.* (2016 ▸) and references therein] despite the fact that SAD phasing is, and has been for quite some time, the method of choice of structure determination if no related structure is available in the Protein Data Bank. In contrast to conventionally collected SAD data where the anomalous correlation CC_ano_ is a good metric for the quality of the anomalous differences, this is rarely the case for SFX data, as also pointed out by Yamashita *et al.* (2017 ▸) in this issue. Thus, better analysis programs are needed urgently, improving SFX data quality such that CC_ano_ (or an alternative measure) becomes meaningful and fewer images (and thus less sample and beam time) are needed to obtain accurate Bragg intensities.

In fact, data processing programs have improved significantly in recent years. This is reflected in the finding that fewer and fewer diffraction patterns of a given dataset are needed for phasing. For example, the very first demonstration of *de novo* phasing required 60000 indexed patterns for fully automatic model building (Barends *et al.*, 2014 ▸), but recently 10000 patterns were sufficient (Nass *et al.*, 2016 ▸). Similarly, two years ago, Yamashita *et al.* (2015 ▸) could not solve their structure using only the anomalous signal of their mercury derivative, and now they can (Yamashita *et al.*, 2017 ▸). The future will show how far this can go but it seems likely that more than the optimization of details is needed before real systems diffracting weakly to medium resolution can be phased with reasonable requirements on sample and beam time.
